# Migrant well-being and undocumented status in South Korea: a cross-sectional assessment of physical, psychological, social well-being, and health behaviors

**DOI:** 10.1186/s12939-024-02126-2

**Published:** 2024-02-26

**Authors:** Sun Yeop Lee, Woong-Han Kim, Jongho Heo

**Affiliations:** 1 JW LEE Center for Global Medicine, Seoul National University College of Medicine, Seoul, 03087 Republic of Korea; 2 Department of Thoracic and Cardiovascular Surgery, Seoul National University College of Medicine, Seoul, 03080 Republic of Korea; 3 National Assembly Futures Institute, Seoul, 07233 Republic of Korea; 4 Department of Human Systems Medicine, Seoul National University College of Medicine, Seoul, 03080 Republic of Korea

**Keywords:** Immigrant, Migrant worker, Undocumented, Health, Physical well-being, Health behavior, Psychological well-being, Social well-being, South Korea

## Abstract

**Background:**

A high burden of physical, mental, and occupational health problems among migrant workers has been well-documented, but data on undocumented migrant workers are limited and their well-being has rarely been compared to that of the general population.

**Methods:**

Using data from a cross-sectional survey of non-professional migrant workers in South Korea in early 2021, we described their physical, psychological, social well-being and health behaviors across a wide range of outcomes, including self-rated health, occupational injury, cigarette smoking, heavy alcohol consumption, meal pattern, happiness, mental illness, social support, and social participation. The outcomes were first compared between documented and undocumented migrant workers in generalized linear regressions adjusting for potential confounders. Then, the well-being of the migrant workers was compared against that of the general population using data from the Korean Happiness Survey, which is a nationally representative survey of the South Korean general population conducted in late 2020. The parametric g-formula was performed to adjust for potential confounders.

**Results:**

After adjusting for potential confounders, the undocumented migrant workers were less likely to be happy or participate in social communities, and much more likely to have anxiety or depression, smoke cigarettes, or engage in heavy alcohol consumption than the documented migrant workers. When compared to the general South Korean population, an evident social gradient emerged for happiness and mental illness; the undocumented experienced the worst outcome, followed by the documented, and then the general population. Also, the undocumented migrant workers were more likely to smoke cigarettes than the general population.

**Conclusion:**

The undocumented migrant workers face considerably greater challenges in terms of mental health and happiness, demonstrate higher rates of risky health behaviors such as smoking and heavy drinking, and experience a lack of social support and community integration. A stark social gradient in happiness, mental illness, and cigarette smoking exists among the documented, undocumented migrant workers and the general population in South Korea. Socio-structural factors are likely to play a crucial role in contributing to the suboptimal level of overall well-being of undocumented migrant workers. Policy-level interventions as well as interpersonal efforts are in urgent need.

**Supplementary Information:**

The online version contains supplementary material available at 10.1186/s12939-024-02126-2.

## Background

As of October 2023, approximately 2.49 million migrants (4.8% of the total population) were estimated to reside in South Korea, which is a large and steady increase from the number of approximately 1.45 million in 2012 [1]. The proportion of those who were undocumented was estimated to be 17.8%, which was also much higher than the 12.3% in 2012. Such an increase is expected to continue, especially for the undocumented portion, and it is closely related to the current labor shortage in South Korea.

According to the International Labour Organization, a migrant worker is “a person who migrates from one country to another with a view to being employed otherwise than on his own account,” excluding those migrate for purposes other than employment [2]. In South Korea, accurately estimating the number of migrant workers is challenging because different governmental departments report on varying scopes of migrant workers, mostly excluding those without the legal status to be employed (“the undocumented migrant worker”). One relatively accurate figure reported that, in 2023, the number of migrants with employment purpose visas was 476,550, of which over 60% were on a non-professional employment visa, E-9 [1]. The main industry sectors for the migrant workers included mining/manufacturing (43.9%), wholesale/accommodation/food services (18.7%), business/personal and public services (16.7%), construction (12.2%), and agriculture/fishery (5.4%) [3], and the nationalities included China (33.3%), Vietnam (17.2%), Nepal (4.4), Uzbekistan (4.0%), and Cambodia (4.0%) [1].

Migrant workers are subject to distinct challenges that affect their health and well-being, such as the migration process, reasons for migrating, length of stay, separation from family, and legal status. Migration itself has been recognized as a social determinant of migrants’ health, with previous research generally focusing on three frameworks: behavioral, cultural, and structural [4, 5]. In addition to traditional social determinants of health such as income, education, and gender, migrant workers face cultural challenges such as social exclusion, stigma, and language barriers. Additionally, they encounter legal and structural challenges in workers’ rights and access to basic services such as healthcare, especially if they are undocumented. They are often involved in dangerous and demanding jobs with occupational hazards and precarious employment status that expose them to high risk of abuse, exploitation, and discrimination. Such health-harming environments are exacerbated for migrant workers without legal status and protection.

Empirically, a number of studies have found that migrant workers have a higher burden of physical, mental, and occupational health problems, compared to their counterparts in host countries [6–9]. They tend to have restricted access to health services [9, 10], and health services may lack cultural sensitivity or present language barriers [11, 12]. In occupational settings, they often face limited employment rights and are forced to take jobs with long working hours and inadequate safety conditions including environmental hazards [6, 13, 14]. For undocumented migrant workers, empirical studies or national surveillance of health are often limited. Many studies have often excluded the undocumented due to legal barriers [6], have been limited to small sample sizes, or have relied on qualitative methods [15].

In South Korea, migrant workers face similar socio-structural discrimination, and such challenges are rooted in the foreign employment systems [16, 17]. The employment systems for foreigners in South Korea are complex, having created and evolved in response to the needs of the industrial structures, such as supplying labor in agriculture and manufacturing. As such, the majority of migrant workers hold the E-9 status under the Employment Permit System and are employed in small and medium-sized manufacturing, construction, service, or agriculture industries. A major issue is that these migrants cannot change their workplaces without their employer’ permission and have limited right to autonomously change their workplaces for at least three years. The unauthorized change in their jobs leads to the undocumented status, leaving the workers vulnerable to exploitation which includes unfairly low wages, long working hours, and poor working conditions [18]. Although undocumented migrants are entitled to report occupational accidents and receive compensation, their reporting rate is lower than that of the documented migrants, highlighting the challenges they face in occupational settings [16]. Regarding policies and programs directly related to health, migrants are generally ineligible for the medical aid program, which are available for low-income South Korean nationals. Undocumented migrants are also not covered by the national health insurance and are left to rely on voluntary services by neighboring clinics or non-profit, non-governmental organizations to meet their medical needs (Koo, 2015). Moreover, the availability of health screening services is limited for migrants and varies greatly by the location of residence [16].

A few empirical studies in South Korea have found that migrant workers have a higher likelihood of experiencing occupational injuries [19–22], long working hours [16], and exposure to environmental hazards [16, 23]. However, reliable data on migrant workers, especially for a wider range of well-being measures, are scarce. In particular, undocumented migrants, more vulnerable to health risks, have often been excluded from or unidentifiable in previous studies, and thus the state of their well-being has rarely been compared to that of documented migrants or the general South Korean population. There is a need for a comprehensive assessment of undocumented and documented migrant workers’ well-being and a reliable comparison to the general population.

In this cross-sectional survey in South Korea, migrant workers’ well-being was measured across a wide range of outcomes. Descriptive statistics on their (1) physical well-being outcomes (self-rated health, occupational injury), (2) health behaviors (cigarette smoking, heavy alcohol consumption), (3) psychological well-being outcomes (happiness, mental illness), and (4) social well-being outcomes (social support, social participation) are presented. Then, we assessed covariate-adjusted associations between the documented status (documented vs. undocumented) and well-being outcomes. Finally, using data from a separate nationally representative cross-sectional survey on the South Korean general population, we compared the well-being of documented and undocumented migrant workers to that of the general population in South Korea.

## Methods

### Data

From February 2021 to March 2021, migrant workers with E-9 (Non-Professional Status) or the undocumented status in South Korea were recruited for a cross-sectional survey. The participants were able to communicate in Korean or their native language, and contacted through officials from migrant worker centers, religious institutions, or Korean language schools in Seoul or Kyunggi-do. A total of 550 individuals were contacted to reach the pre-determined sample size of 500 with the potential non-response rate of 10% according to prespecified quotas by documented status (1:1 = documented:undocumented), gender (1:1 = female:male), and employment status (3:7 = unemployed:employed). Those who were undocumented were oversampled to obtain reliable estimates. The survey was originally carried out to gain an understanding of migrant workers’ health literacy and healthcare access regarding COVID-19. The participants filled out written consent forms and completed in-person, self-report questionnaires in Korean or their native languages (Nepali, Russian, Vietnamese, English, Uzbek, Thai, or Tagalog). The questionnaires collected information on the participants’ documented status, employment status and working conditions, physical and psychological well-being, health-related experiences, social well-being, and health literacy regarding COVID-19. Following the completion of the questionnaires, the participants were compensated with 10,000 South Korean won (KRW).

We also utilized data from the Koreans’ Happiness Survey (KHS), a nationally representative cross-sectional survey of citizens aged 15 or older [24]. The survey was conducted from October to December 2020, matching the time period of the migrant worker survey. The survey employed a stratified cluster sampling method to ensure representativeness and collected information to describe the happiness level and its inequality in South Korea. Further details of the survey including its sample characteristics have been described elsewhere [25].

### Dependent variables

A variety of questions were asked to assess the physical, psychological, and social well-being and health behaviors of the migrant workers during their time residing in South Korea. These variables were analyzed after some modifications for greater statistical power or better interpretability.

For physical well-being outcomes, the participants were asked about current self-rated health (very good, fairly good, average, fairly bad, very bad) and their experiences of an occupational injury (yes, no) during their time in South Korea. The self-rated health was dichotomized following the common practice [26]. For health behaviors, they were asked about their current smoking status (yes, no, in the past), per-day amount of smoking (< 10, 11–20, 21–30, > 31), per-week frequency of alcohol consumption (none, 1–2, more than three, only in the past), per-occasion amount of alcohol consumption (1, 2, 3, 4–5, > 6), and meal patterns (regularly three meals a day, sometimes skipping a meal, often irregular). A binary variable of current smoking status was generated by regrouping those who answered “no” and “in the past” into a single group of currently not smoking. A categorical variable of alcohol consumption level (none, moderate, heavy) was generated by classifying those with 4 drinks or more as heavy drinking and less as moderate drinking based on the definition by the National Institute on Alcohol Abuse and Alcoholism [27]. A binary variable of irregular meal patterns was generated by regrouping those who regularly had three meals a day or sometimes skipped a meal as a single group of regular meal patterns.

Psychological well-being outcomes included a general level of happiness (0–10), Generalized Anxiety Disorder-2 (GAD-2; less than one day per week, one to two days per week, three to four days per week, more than five days per week), and Patient Health Questionnaire-2 (PHQ-2; the same scale as GAD-2). The GAD-2 measures a psychological state of anxiety or worry, while the PHQ-2 measures a psychological state of apathy or depression. Both have been reported to have great internal consistency (Cronbach’s $\alpha$ = 0.86, 0.81 for GAD-2, PHQ-2, respectively) [28, 29]. Per previous recommendations, GAD-2 and PHQ-2 were analyzed as binary variables with a threshold of score 3 ( > = 3, < 3) [30, 31]. Large meta-analyses showed that at the threshold of score 3, the GAD-2 had sensitivity of 0.76 and specificity of 0.81 for screening the GAD [32], and the PHQ-2 had sensitivity of 0.72 and specificity of 0.85 for screening for major depression [33]. In addition, the total four questions in the GAD-2 and PHQ-2 were summed to calculate a mental illness score, and this outcome was treated as continuous.

For social well-being, questions on social support and social participation were asked. Social support questions asked whether a participant had a person to talk to when lonely or a person to ask for help when in trouble (strongly agree, agree, disagree, strongly disagree). These two variables were dichotomized into agreeing or disagreeing with the questions. Social participation questions asked whether interviewees participated in an ethnic, recreational, or religious community (never, in the past, sometimes, actively, very actively). Due to small sample size, these variables were dichotomized into having never participated or ever participated, which was consistent with several previous studies [34–36].

### Independent variables

Immigration status was recorded by choosing one of three options: E-9 (non-professional employment), undocumented, or others. Others included visa types other than E-9, the most common work visa for immigrants in South Korea. The immigrant status variable was dichotomized for analyses (documented, undocumented).

A range of sociodemographic variables was selected as potential confounders for associations between immigrant status and well-being outcomes. The sociodemographic variables included age (months), gender (male, female), location of residence, months residing in South Korea, monthly income (< 1 million KRW, 1–1.5 million KRW, 1.5–2 million KRW, > 2 million KRW), employment status (employed, unemployed), industry sector (mining/manufacturing, agriculture/forestry/fishing, construction, wholesale/retail/lodging/food business, electricity/transportation/communication/finance, self-employed, public service, domestic helper, others), educational attainment (elementary school, middle school, high school, college, graduate school), house size (one bedroom & one bathroom, two bedrooms & one bathroom, two bedrooms & two bathrooms, three bedrooms & two bathrooms or larger), housing quality on structural safety, water resistance, insulation, ventilation, natural light, security, sanitation (poor, slightly poor, slightly good, good), number of cohabitants, marital status (partner in Korea, partner not in Korea, divorced, widowed, unmarried), country of origin, and religion (Protestant, Catholic, Buddhist, Muslim, others, none).

Some modifications were applied before entering statistical analyses to avoid bias due to small sample size (e.g., sampling zeros causing unstable estimates) or enhance interpretability. Age in months was calculated from a birth date, assuming that each participant completed the survey in March 2021. Educational attainment levels were regrouped as a four-category variable (middle school or less, high school graduate, college or above, no response). Industry sector categories were regrouped as a four-category variable (mining/manufacturing, agriculture/forestry/fishing, construction, others). For marital status, those who were widowed or divorced were grouped as one category. House size categories were regrouped to create a binary variable of living in a studio (a single room house) or a two-room house (no participant lived in a house with three rooms or more). Housing quality questions were scored as 1–4 for poor, slightly poor, slightly good, and good, and then combined to generate a housing quality score.

### Statistical analyses

Descriptive characteristics of the migrant worker survey were presented for the overall sample, and separately for the documented and undocumented immigrants. Mean or proportion differences across the documented status were tested with the two-sample t-test for normal continuous variables, the Kruskal-Wallis test for non-normal continuous variables, and the chi-square test for categorical variables. There were no missing data for all analyzed variables, except for educational attainment (3.41%) and marital status (1.52%). In all following analyses, the missing data were treated by a simple approach of including a missing indicator for each of these variables because their impact was expected to be negligible. All analyses were performed in R 4.1.1 [37].

To assess associations between documented status and well-being outcomes, a linear regression was performed for continuous outcomes, a logistic regression was performed for binary outcomes, and a multinomial logistic regression was performed for categorical outcomes. For the primary analyses, all independent variables listed above were included in the regressions to adjust for potential confounding, except for the industry sector variable. This variable causes a sampling zero when intersected with the documented status, which may result in unstable estimates. Therefore, regressions including this variable were performed as supplementary. In addition, due to the cross-sectional nature of the data, outcomes could be confounders for each other. Therefore, regressions were also performed adjusting for outcomes of other well-being categories as sensitivity analyses (e.g., when analyzing psychological well-being outcomes, outcomes of physical and social well-being and health behaviors were included in the regressions). Furthermore, to check robustness of the decisions in the primary analyses to re-categorize outcomes to avoid sampling zeros and enhance interpretability, another set of regressions were performed using the original (thus, finer) forms of outcome categorization. Sampling zeros caused failures to model convergence for the meal pattern variable and the social participation variables; the meal pattern variable was excluded from this sensitivity analysis, while the social participation variables were regrouped into three categories (no participation, former participation, active participation). Firth’s bias correction was applied to logistic regressions and multinomial regressions to reduce bias from separation, which may occur due to a small sample size and a relatively large list of covariates to control [38]. For binary or categorical outcomes, E-values were calculated to evaluate the robustness of observed associations to unmeasured confounding [39, 40]. A square root transformation was applied under the common outcome assumption. An E-value assesses the minimum strength of association that an unmeasured confounder would have to have on the risk ratio scale with both the exposure and a given outcome to explain away the association. Multiple testing was taken into account by controlling the false discovery rate with the Benjamini-Hochberg procedure at a threshold of 0.05 [41]. Uncorrected *p* values and 95% confidence intervals were also presented for clearer interpretation without any arbitrary correction.

To compare the well-being of migrant workers to that of the general population, we pooled the individual-level data of the migrant worker survey data and the KHS data. We restricted the KHS data to those aged over 19 and under 62 to match the age range of the migrant worker survey data, avoiding positivity violation and extrapolation beyond the data range. We performed the parametric g-formula to compare well-being outcomes across documented migrant workers, undocumented migrant workers, and the general population. The parametric g-formula, which is also called a regression-based standardization, was set up in a way that it estimates what outcomes would be if each group had the same covariate distributions as the reference group (i.e., the general population) [42]. We adjusted for age, gender, education, employment status, marital status. Other covariates used in the analyses of the migrant worker survey could not be included here because they were either not collected in the KHS data or collected in a different form. We were also only able to analyze health behaviors and psychological well-being because other well-being outcomes were not available in the KHS data or collected in an incomparable form. The alcohol consumption outcome was treated as a binary variable of drinker vs. non-drinker because, in the KHS, the level of alcohol consumption was recorded as a numerical response question, as opposed to a multiple-choice question in the migrant worker survey, and had over 80% missing data. It is important to note that the estimates for each sample were not representative of any larger populations, but only used to make between-group comparisons.

## Results

**Table 1:** Sample characteristics of migrant workers in South Korea

	Overall	Documented	Undocumented	*p*
Total	528	278	250	
Age (mean)	34.30 (5.68)	34.23 (6.40)	34.37 (4.78)	0.780
Female (%)	262 (49.62)	131 (47.12)	131 (52.40)	0.261
Months in Korea (IQR)	43.00 [29.00, 73.00]	32.00 [25.00, 48.00]	69.00 [40.00, 76.00]	< 0.001
Unemployed (%)	156 (29.55)	74 (26.62)	82 (32.80)	0.145
Industry sector (%)				< 0.001
Mining / manufacturing	250 (47.35)	190 (68.35)	60 (24.00)	
Agriculture / forestry / fishing	23 (4.36)	10 (3.60)	13 (5.20)	
Construction	28 (5.30)	4 (1.44)	24 (9.60)	
Others	71 (13.45)	0 (0.00)	71 (28.40)	
Income (%)				< 0.001
1 million KRW <	16 (3.03)	0 (0.00)	16 (6.40)	
1–1.5 million KRW	116 (21.97)	32 (11.51)	84 (33.60)	
1.5–2 million KRW	180 (34.09)	129 (46.40)	51 (20.40)	
2 million KRW >	60 (11.36)	43 (15.47)	17 (6.80)	
Educational attainment (%)				0.036
College or above	153 (28.98)	90 (32.37)	63 (25.20)	
High school	347 (65.72)	172 (61.87)	175 (70.00)	
Middle school	20 (3.79)	14 (5.04)	6 (2.40)	
Not answered	8 (1.52)	2 (0.72)	6 (2.40)	
House size (%)				0.008
One bedroom	371 (70.27)	205 (73.74)	166 (66.40)	
Two bedrooms / one bathroom	140 (26.52)	70 (25.18)	70 (28.00)	
Two bedrooms / two bathrooms	17 (3.22)	3 (1.08)	14 (5.60)	
Housing quality score (mean)	16.67 (2.73)	17.10 (2.47)	16.20 (2.94)	< 0.001
Number of cohabitants [IQR]	2.00 [1.00, 3.00]	2.00 [1.00, 2.00]	2.00 [2.00, 3.00]	< 0.001
Marital status (%)				0.144
Married (partner in Korea)	29 (5.49)	12 (4.32)	17 (6.80)	
Married (partner not in Korea)	197 (37.31)	93 (33.45)	104 (41.60)	
Divorced	4 (0.76)	1 (0.36)	3 (1.20)	
Widowed	10 (1.89)	6 (2.16)	4 (1.60)	
Unmarried	270 (51.14)	155 (55.76)	115 (46.00)	
Not answered	18 (3.41)	11 (3.96)	7 (2.80)	

Continuous variables were presented as mean (standard deviation), except nonnormal variables (months in Korea, number of cohabitants), which were presented as median (interquartile range). Categorical variables were presented as count (percentage)

*KRW *South Korean Won, *IQR *Interquartile range

The distributions for immigration, gender, and employment status were close to the pre-assigned quotas in sampling (Table 1); there were 278 documented (52.65%) and 250 undocumented migrants (47.35%), 262 females (49.62%) and 266 males (50.38%), and 156 unemployed (29.55%) and 372 employed individuals (70.45%). The mean age in the overall sample was 34.30 (SD = 5.68, range = [19.90, 61.00]). On average, the age and gender were comparable across the documented status (*p* = 0.780 and *p* = 0.261, respectively). Within this sample, the undocumented migrants, on average, had resided in South Korea longer than the documented migrants (32 vs. 69 months; *p* < 0.001). Compared to the documented, the undocumented were less likely to earn higher than 2 million KRW (6.8% vs. 15.47%; *p* < 0.001), have completed education at the college level or above (25.20% vs. 32.37%; *p* < 0.036), and live in a one-bedroom place (66.40% vs. 73.74%; *p* = 0.008). Countries of origin (n) included Thailand (53; 10.0%), Nepal (52; 9.8%), Philippines (67; 12.7%), Vietnam (104; 19.7%), Uzbekistan (68; 12.9%), Cambodia (62; 11.7%), Indonesia (59, 11.2%), Kazakhstan (11; 2.1%), Bangladesh (30; 5.7%), Mongolia (14; 2.7%), and others (8; 1.5%). The participants’ religions included Protestant (10; 1.9%), Catholic (40; 7.6%), Buddhist (108; 20.5%), Muslim (107; 20.3%), and Hindu (52; 9.8%), and 211 (40.0%) did not associate with any religion. The sociodemographic characteristics and outcomes of the KHS study sample of the general population in South Korea are presented in Supplementary Table 1.

**Table 2:** Unadjusted comparisons of well-being outcomes by the documented status

	Overall	Documented	Undocumented	*p*
Self-rated health (%)				0.004
Very good	159 (30.11)	101 (36.33)	58 (23.20)	
Fairly good	326 (61.74)	158 (56.83)	168 (67.20)	
Average	43 (8.14)	19 (6.83)	24 (9.60)	
Occupational injury (%)	48 (9.09)	15 (5.40)	33 (13.20)	0.003
Cigarette smoking (%)	124 (23.48)	55 (19.78)	69 (27.60)	0.036
Never-smoker	387 (73.30)	218 (78.42)	169 (67.60)	
Former smoker	17 (3.22)	5 (1.80)	12 (4.80)	
10 or less/day	61 (11.55)	28 (10.07)	33 (13.20)	
11–20/day	50 (9.47)	23 (8.27)	27 (10.80)	
21–30/day	13 (2.46)	4 (1.44)	9 (3.60)	
Alcohol consumption level (%)				0.086
Non-drinker	249 (47.16)	141 (50.72)	108 (43.20)	
Moderate	261 (49.43)	131 (47.12)	130 (52.00)	
Heavy	18 (3.41)	6 (2.16)	12 (4.80)	
Meal pattern (%)				< 0.001
Regular (three meals/day)	144 (27.27)	106 (38.13)	38 (15.20)	
Sometimes skip a meal	259 (49.05)	124 (44.60)	135 (54.00)	
Irregular (always skip one or more meals)	125 (23.67)	48 (17.27)	77 (30.80)	
Happiness (mean)	5.78 (1.36)	6.14 (1.29)	5.38 (1.32)	< 0.001
GAD-2 > = 3 (%)	155 (29.36)	40 (14.39)	115 (46.00)	< 0.001
PHQ-2 > = 3 (%)	124 (23.48)	32 (11.51)	92 (36.80)	< 0.001
Having a person to talk to when lonely (%)				0.002
Strongly disagree	17 (3.22)	2 (0.72)	15 (6.00)	
Disagree	101 (19.13)	47 (16.91)	54 (21.60)	
Agree	386 (73.11)	216 (77.70)	170 (68.00)	
Strongly agree	24 (4.55)	13 (4.68)	11 (4.40)	
Having a person to ask for help when in trouble (%)				
Strongly disagree	18 (3.41)	3 (1.08)	15 (6.00)	0.014
Disagree	143 (27.08)	76 (27.34)	67 (26.80)	
Agree	335 (63.45)	179 (64.39)	156 (62.40)	
Strongly agree	32 (6.06)	20 (7.19)	12 (4.80)	
Ethnic community participation (%)				
Never	206 (39.02)	94 (33.81)	112 (44.80)	0.120
Formerly	150 (28.41)	83 (29.86)	67 (26.80)	
Sometimes	125 (23.67)	72 (25.90)	53 (21.20)	
Actively	44 (8.33)	27 (9.71)	17 (6.80)	
Recreational community participation (%)				< 0.001
Never	447 (84.66)	215 (77.34)	232 (92.80)	
Formerly	57 (10.80)	45 (16.19)	12 (4.80)	
Sometimes	20 (3.79)	16 (5.76)	4 (1.60)	
Actively	4 (0.76)	2 (0.72)	2 (0.80)	
Religious community participation (%)				
Never	319 (60.42)	165 (59.35)	154 (61.60)	0.306
Formerly	86 (16.29)	52 (18.71)	34 (13.60)	
Sometimes	79 (14.96)	36 (12.95)	43 (17.20)	
Actively	41 (7.77)	24 (8.63)	17 (6.80)	
Very actively	3 (0.57)	1 (0.36)	2 (0.80)	

Continuous variables were presented as mean (standard deviation), except nonnormal variables (months in Korea, number of cohabitants), which were presented as median (interquartile range). Categorical variables were presented as count (percentage)

*GAD *Generalized Anxiety Disorder, *PHQ *Patient Health Questionnaire

Unadjusted comparisons showed that the state of health and well-being of the migrant workers greatly differ by the documented status (Table 2). The undocumented reported worse self-rated health (*p* < 0.004) and more occupational injuries (*p* = 0.003), compared to the documented. The undocumented were also more likely to be smokers (*p* = 0.036) and have irregular meal patterns (*p* < 0.001). In terms of psychological well-being, the undocumented scored lower on happiness (*p* < 0.001), or three or higher on GAD-2 (*p* < 0.001) or PHQ-2 (*p* < 0.001). In addition, compared to the documented, the undocumented were less likely to have a person to talk when lonely (*p* = 0.002) or ask for help when in trouble (*p* = 0.014), and less likely to participate in recreational communities (*p* < 0.001).

**Table 3:** Associations of the undocumented status with well-being outcomes after adjusting for potential confounders

	Undocumented vs. Documented				
	OR (95% CI)	$\beta$(95% CI)	*p*-value	FDR-adjusted *p*-value	*E* value
*Physical well-being*					
Self-rated health (average or worse)	1.43 (0.62, 3.29)	-	0.398	0.427	1.68
Occupational injury	2.03 (0.93, 4.46)	-	0.077	0.105	2.20
*Health behavior*					
Cigarette smoking	2.05 (1.11, 3.78)	-	0.022	0.047*	2.22
Alcohol consumption level					
– moderate vs. none	1.52 (0.94, 2.46)	-	0.086	0.105	1.77
– heavy vs. none	5.66 (1.82, 17.57)	-	0.003	0.041*	4.19
Irregular meal pattern	1.77 (1.05, 3.01)	-	0.034	0.056	2.00
*Psychological well-being*					
Happiness	-	-0.65 (-0.89, -0.40)	< 0.001	< 0.001*	-
GAD-2 > = 3	4.99 (2.79, 8.92)	-	< 0.001	< 0.001*	3.89
PHQ-2 > = 3	3.40 (1.84, 6.26)	-	< 0.001	< 0.001*	3.09
Mental illness score	-	1.57 (1.11, 2.03)	< 0.001	< 0.001*	-
*Social well-being*		-			
Having a person to talk to when lonely	0.84 (0.47, 1.53)	-	0.576	0.576	1.40
Having a person to ask for help when in trouble	0.78 (0.46, 1.32)	-	0.358	0.413	1.51
Recreational community participation	0.20 (0.10, 0.41)	-	< 0.001	< 0.001*	3.92
Ethnic community participation	0.38 (0.22, 0.67)	-	< 0.001	0.002*	2.63
Religious community participation	0.53 (0.29, 0.96)	-	0.038	0.056	2.09

All regressions were adjusted for age, gender, location of residence, months residing in Korea, monthly income, employment status, educational attainment, house size, housing quality, number of cohabitants, marital status, country of origin, and religion. Uncorrected p values were presented, while statistical significance after Benjamini-Hochberg false discovery rate correction was denoted with an asterisk (*)

*OR *Odds ratio, *CI *Confidence intervals, *GAD *Generalized Anxiety Disorder, *PHQ *Patient Health Questionnaire, *FDR *False discovery rate

After adjusting for potential confounders and applying multiple testing correction, being undocumented was associated with worse outcomes across a range of health behaviors, and psychological and social well-being, compared to being documented (Table 3). The undocumented migrant workers had lower levels of happiness ($\beta$ [95% CI] = -0.65 [-0.89, -0.40]; *p* < 0.001), higher levels of the mental illness score ($\beta$ [95% CI] = 1.57 [1.11, 2.03]; *p* < 0.001), higher odds of scoring three or higher on the GAD-2 (OR [95% CI] = 4.99 [2.79, 8.92]; *p* < 0.001) and the PHQ-2 (OR [95% CI] = 3.40 [1.84, 6.26]; *p* < 0.001), when compared to the documented migrant workers. The undocumented were more likely to be current smokers (OR [95% CI] = 2.05 [1.11, 3.78]; *p* = 0.022) and current heavy alcohol drinkers (OR [95% CI] = 5.66 [1.82, 17.57]; *p* = 0.003). They were also found to be less likely to ever participate in an ethnic community (OR [95% CI] = 0.38 [0.22, 0.67]; *p* < 0.001) and a recreational community (OR [95% CI] = 0.20 [0.10, 0.41]; *p* < 0.001). These eight associations had E-values ranging from 2.22 to 4.19, suggesting that these associations were moderately robust to unmeasured confounding. For example, an unmeasured confounder associated with both being undocumented and scoring high on GAD-2 by risk ratios of 3.89 each, above and beyond the adjusted covariates, could suffice to explain away the association, but weaker confounding could not. The associations for the eight outcomes remained statistically significant after additionally adjusting for industry sectors (Supplementary Table 2) or outcomes of other well-being categories (Supplementary Table 3), demonstrating the robustness to model misspecifications. No statistically significant patterns were found by the documented status for self-rated health, occupational injury, irregular meal pattern, having a person to talk to when lonely or a person to ask for help when in trouble. When finer categorization of outcomes was adopted, the findings were consistent except now that some statistically significant patterns were found for the undocumented being less likely to have a person to talk to when lonely or a person to ask for help when in trouble, compared to the documented (Supplementary Table 4).

**Fig. 1:**
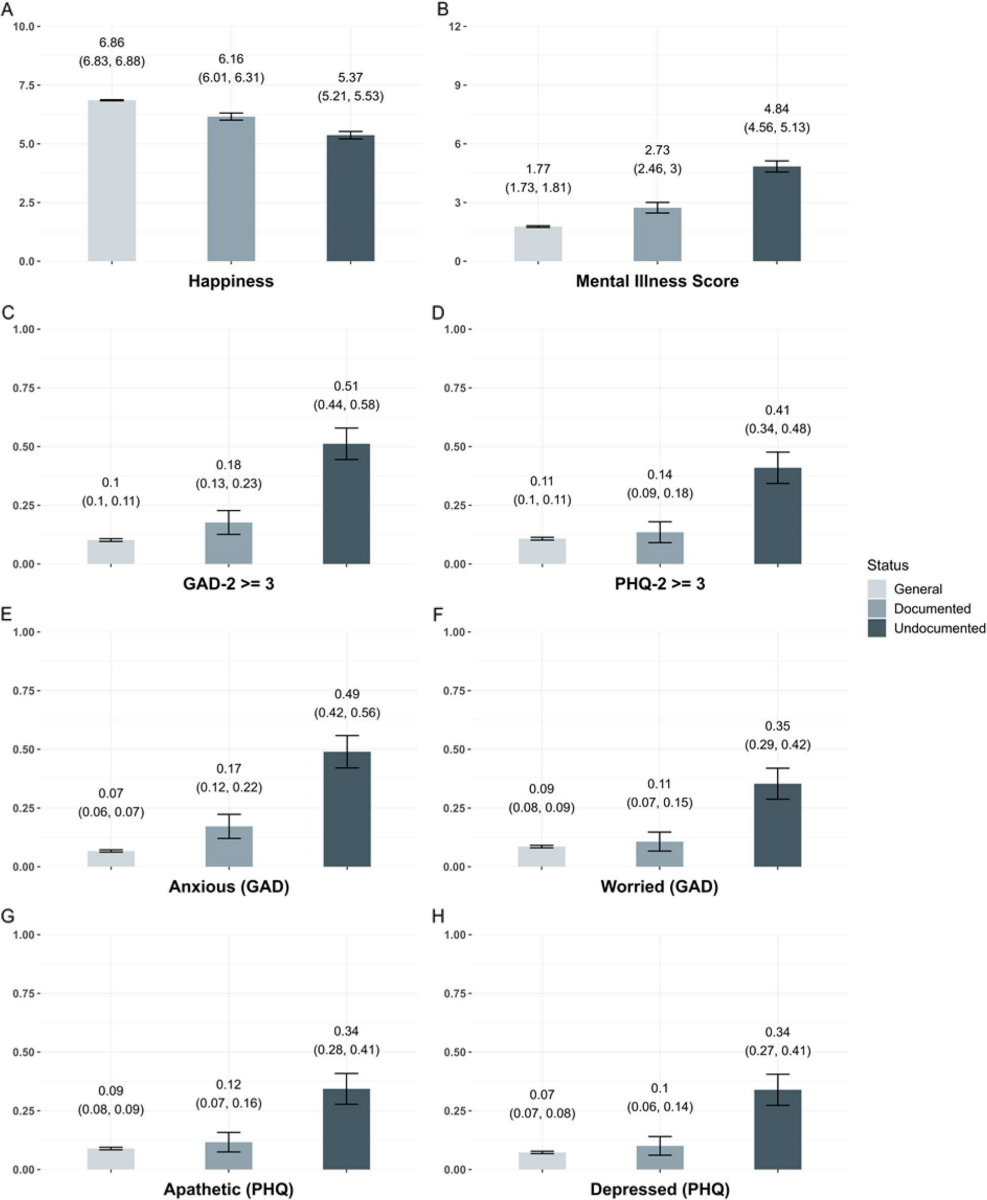
Covariate-standardized comparisons of happiness and mental health outcomes across the general population, documented migrant workers and undocumented migrant workers

The measures of happiness, mental illness, cigarette smoking, and alcohol consumption were also measured in the KHS data, enabling comparisons to the general population. The parametric g-formula analyses adjusting for a range of covariates found that the mean happiness levels [95% CI] were the lowest for the undocumented migrant workers (5.37 [5.21, 5.53]), followed by the documented migrant workers (6.16 [6.01, 6.31]), and then the general population (6.86 [6.83, 6.88]) (Fig. 1). The same social gradient was found for the mean mental illness score with the worst outcome experienced by the undocumented migrant workers (mean [95% CI] = 4.84 [4.56, 5.13]). This gradient for the mental illness score may be driven by the GAD-2 as the same pattern was observed for the GAD-2, but not for the PHQ-2; while the difference in probabilities [95% CI] of scoring high on the PHQ-2 between the general population (0.11 [0.10, 0.11]) and the documented migrant workers (0.14 [0.09, 0.18]) was not statistically significant, but it was three- to four-fold for the undocumented migrant workers (0.41 [0.34, 0.48]). When investigated in detail by analyzing individual questions in the GAD-2 and PHQ-2, the undocumented migrant workers consistently had the worst outcomes for all four questions, while only the question on feeling anxiety had the same statistically significant social gradient found above. However, in point estimates, all other questions also had the same social gradient.

**Fig. 2:**
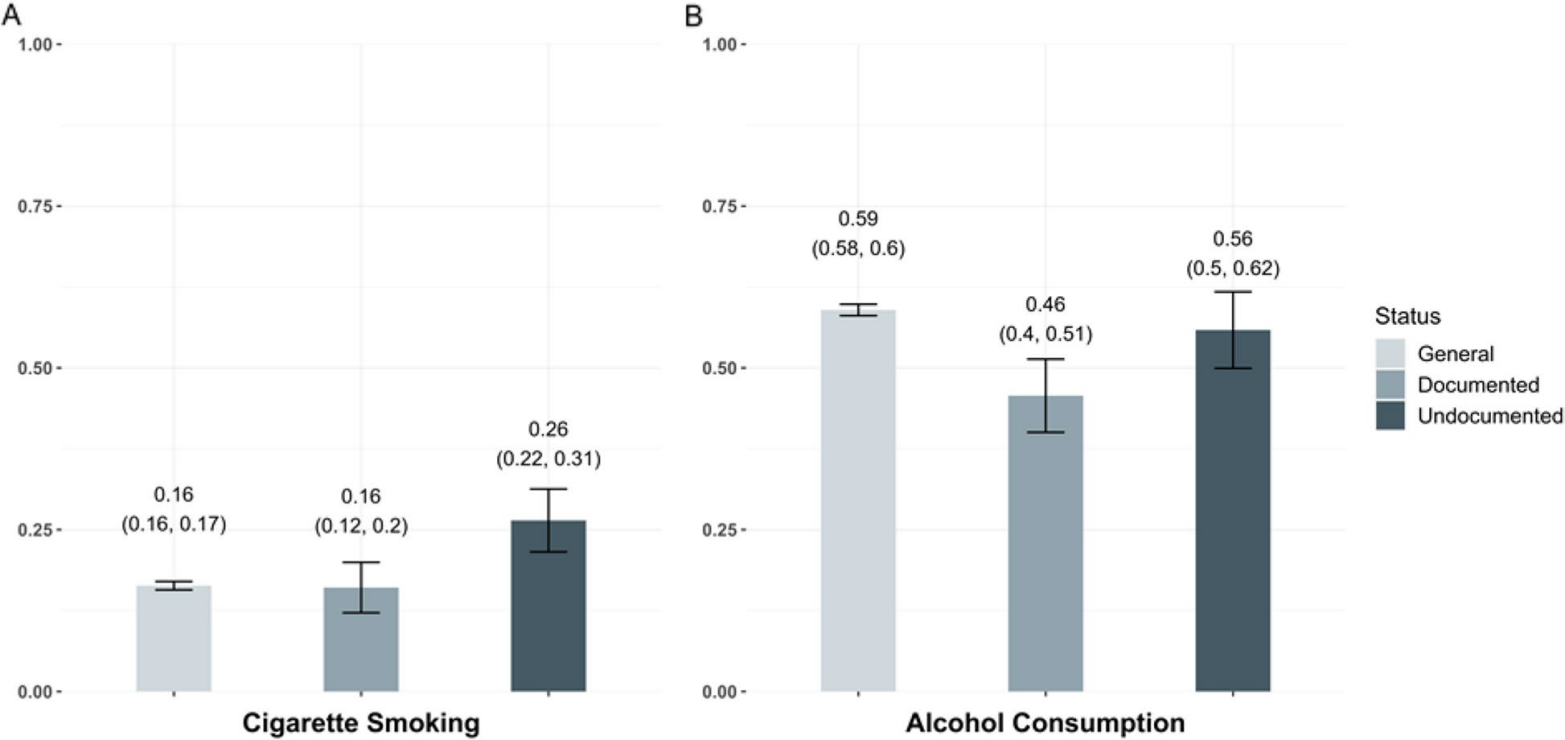
Covariate-standardized comparisons of cigarette smoking and alcohol consumption across the general population, documented migrant workers and undocumented migrant workers

For smoking and alcohol consumption, a slightly different pattern was observed (Fig. 2). For being a current smoker, undocumented migrant workers were most likely (probability [95% CI] = 0.26 [0.22, 0.31]), and the estimates were similar for the general population (probability [95% CI] = 0.16 [0.16, 0.17]) and the documented migrant workers (probability [95% CI] = 0.16 [0.12, 0.20]). For being a current alcohol drinker, the documented migrant workers were least likely (probability [95% CI] = 0.46 [0.40, 0.51]), while the estimates were higher for the general population (probability [95% CI] = 0.59 [0.58, 0.60]) and the undocumented migrant workers (probability [95% CI] = 0.56 [0.50, 0.62]).

## Discussion

Using data from a survey of migrant workers residing in South Korea, we described the state of their physical, psychological, social well-being and health behaviors across a range of measures. After adjusting for several sociodemographic variables, we found that there were inequalities of considerable magnitude in well-being outcomes. Compared to the documented, the undocumented migrant workers were less likely to be happy or participate in social communities. They also showed higher tendencies towards anxiety, depression, cigarette smoking, or heavy alcohol use. When compared to the general South Korean population, an evident social gradient emerged, particularly in psychological well-being, with the undocumented faring the worst, followed by the documented, and then the general population.

The most pronounced inequality by documented status was observed in psychological well-being. There have been a couple of reports in South Korea of migrant workers’ mental health presenting in similar levels to native workers [16, 43], whereas one study found that a very high proportion (approximately 70%) of undocumented, male migrant workers had depressive symptoms [44]. Adding to these scattered pieces of evidence, we found, in a single reliable data source, that the disparity in mental health by documented status was substantial. Bringing the general population estimates into the picture, a clear social gradient was observed. Not only were the undocumented migrant workers at the worst psychological states across all the measured outcomes, but the documented migrant workers also fared worse than the general population, creating a social gradient from the undocumented migrants to documented migrants to the general population. Previously identified factors associated with the psychological well-being of migrant workers in South Korea include linguistic barriers, cultural difficulties, social isolation, interpersonal discrimination, and female gender [45–47]. In addition, those with undocumented status lack sufficient legal protections and are likely at a higher risk of exploitation [18]. A qualitative survey also revealed that the undocumented migrant workers live in constant fear of surveillance and deportation, which cause psychiatric issues, but the infrastructure to address these issues is scarce [16].

Undocumented migrant workers were less likely to participate in social communities, such as ethnic and recreational communities, but they did not differ in their likelihood of having a person to talk when lonely and ask for help when in trouble. While undocumented migrants may have intimate social connections, like direct family members, they may not feel safe expanding their social networks to wider communities due to their legal status and fear of deportation or discrimination. Positive impact of aspects of social capital, such as social participation and civic engagement, on health has long been recognized in a general setting [48–50] and in the context of migrant health [51–53]. Also, in South Korea, migrant workers’ physical and psychological well-being were found to be positively influenced by social support and community participation [45, 47, 54, 55]. Accordingly, the lower level of social participation by the undocumented observed in this study may partially explain their worse psychological well-being.

The undocumented migrant workers were also more likely to engage in cigarette smoking and heavy alcohol consumption than the documented migrant workers. Compared to the general population, the prevalence of smoking was higher, while the prevalence of alcohol consumption was comparable.

Our findings on the suboptimal states of migrant workers’ well-being should prompt policy-level actions. While documented migrants are covered by the South Korean national health insurance, they are mostly not eligible for the medical aid, another primary social welfare program for medical services available for low-income South Korean nationals. The national health insurance does not cover undocumented migrants, leaving them without reliable access to the healthcare system. Non-profit, non-governmental organizations provide medical services for the undocumented migrants but the source of funding is not stable in long-term. Related issues such as linguistic and cultural difficulties in communicating with medical professionals, limited access to health-related information, and lack of personal caregivers further complicate their health management and leads to infringement on their right to health [9–12, 17, 56, 57]. The findings of this study especially warrant special attention to the psychological well-being of migrant workers. Those with the undocumented status, in particular, experience concerningly low level of psychological well-being. The foreign employment systems in South Korea, which has been pinpointed as the fundamental, structural cause of migrant health inequalities in South Korea, must be addressed to ensure the migrants’ right to healthcare, the empowerment of migrants at workplace, and minimize the flow of migrants to the undocumented status.

This study has limitations. First, although the migrant worker survey was self-administered and not interviewer-administered, social desirability bias may still have influenced the responses [58]. Migrant workers, who are constantly under the pressure of being a “desirable worker”, might tend to overstate their well-being such as being overly generous to their self-rated health or not admitting to having had an occupational injury. However, the bias would be greater for the undocumented migrants as they are at risk of deportation, and this suggests our findings would be conservative estimates. Second, migrant workers with undocumented status are often underrepresented in population studies. They may be less likely to spare time for data collection compared to those with documented status or may feel unsafe to participate without legal protections. Selection bias is likely to be present, but it would bias towards the null, resulting in conservative estimates; the undocumented migrants in the study are likely to be healthier than those who are not in the study. Given the surveys took place in locations such as migrant worker centers, religious institutions, and Korean language schools, it can be argued that the participants would be more likely to fare worse in economic conditions as well as health and well-being than non-participants. However, even if this were true, the undocumented migrants would be also more likely to participate, resulting in the bias with the same direction (towards the null). Nonetheless, when selecting into the study sample depends on the participants themselves as in this study, the true direction of the bias is difficult to assess, and the caution is warranted when interpreting the results. Second, while the general population in the KHS was nationally representative, the migrant worker survey was not. Despite the standardization over the covariates, residual confounding may be present. Third, while the sample size was large compared to previous studies, they did not allow for comparisons among intersectional groups, such as female undocumented migrant workers. Intersectionality theory has been suggested as an important lens through which to understand migrant health both theoretically and empirically (Evans and Erickson [59]; Kern et al. [60]; Viruell-Fuentes et al. [61]). For example, in South Korea, female undocumented migrant workers face additional challenges in receiving maternity leave, sexual harassment and assault, or lower wages compared to the male counterparts (Jang et al. [62]). Finally, the cross-sectional data cannot rule out residual confounding, reverse causation, or incorrectly adjusting for mediators. However, our sensitivity analyses adjusting for outcomes of other well-being categories showed consistent results. Also, longitudinal studies on undocumented migrants may be unrealistic to conduct due to their high likelihood of dropping out.

This study described the current state of migrant workers’ well-being in South Korea, highlighting significant disparities, especially among those with undocumented status. Our findings revealed that, compared to the documented migrant workers, the undocumented migrant workers face considerably greater challenges in terms of mental health and happiness, demonstrate higher rates of risky health behaviors such as smoking and heavy drinking, and experience a lack of social support and community integration. In particular, outcomes such as happiness and social isolation are not medically defined and cannot be solely explained by individual-level factors such as health behaviors or biomedical factors such as healthcare quality and access. Socio-structural factors are likely to play a crucial role in contributing to the psychological distress and social isolation of the undocumented migrant workers. Policy-level interventions as well as interpersonal efforts are in urgent need to improve undocumented migrant workers’ overall well-being and health behaviors.

### Supplementary Information

**Supplementary Material 1.**

## Data Availability

The migrant worker survey data are available from the corresponding author on reasonable request. The KHS data are publicly available (https://kossda.snu.ac.kr/handle/20.500.12236/25493).

## Abbreviations

KHS: Korean Happiness Survey
GAD-2: Generalized Anxiety Disorder-2
PHQ-2: Patient Health Questionnaire-2
KRW: South Korean won
OR: Odds ratio
CI: Confidence interval
