# Supplementary material for: Migrant well-being and undocumented status in South Korea: a cross-sectional assessment of physical, psychological, social well-being, and health behaviors
Source: Int J Equity Health. 2024 Feb 26;23:38. doi: 10.1186/s12939-024-02126-2 (PMC10895811; doi:10.1186/s12939-024-02126-2)
Supplement: Supplementary file 1 — Supplementary Material 1. [file 12939_2024_2126_MOESM1_ESM.docx]

**Supplementary Table 1** Sample characteristics of the general population study sample (19<age<62) in South Korea

|  | Overall |
| --- | --- |
| Total | 10467 |
| Age* (mean) | 43.69 (12.04) |
| Female (%) | 5577 (53.28) |
| Unemployed (%) | 3013 (28.79) |
| Educational attainment (%) |  |
| College or above | 6957 (66.47) |
| High school | 3267 (31.21) |
| Middle school | 219 (2.09) |
| No answer | 24 (0.23) |
| Cigarette smoking (%) | 1709 (16.33) |
| Alcohol consumption level (%) | 6170 (58.95) |
| Happiness (mean) | 6.86 (1.24) |
| GAD-2 >= 3 (%) | 1036 (10.16) |
| PHQ-2 >= 3 (%) | 1122 (10.72) |
| Note.  *the age range of the general population was restricted to match the age range of the migrant workers.  Continuous variables were presented as mean (standard deviation). Categorical variables were presented as count (percentage).  GAD, Generalized Anxiety Disorder; PHQ, Patient Health Questionnaire. | |

**Supplementary Table 2** Associations of the undocumented status with well-being outcomes after adjusting for potential confounders including industry sectors

|  | Undocumented vs. Documented | | |
| --- | --- | --- | --- |
|  | OR (95% CI) | $\beta$ (95% CI) | p-value |
| *Physical well-being* |  |  |  |
| Self-rated health (average or worse) | 1.49 (0.40, 5.60) | - | 0.556 |
| Occupational injury | 1.23 (0.39, 3.88) | - | 0.719 |
|  |  |  |  |
| *Health behavior* |  |  |  |
| Cigarette smoking | 1.46 (0.57, 3.74) | - | 0.430 |
| Alcohol consumption level |  |  |  |
| – moderate vs. none | 1.93 (0.90, 4.16) | - | 0.092 |
| – heavy vs. none | 10.49 (1.61, 68.51) | - | 0.014 |
| Irregular meal pattern | 2.73 (1.10, 6.75) | - | 0.030 |
|  |  |  |  |
| *Psychological well-being* |  |  |  |
| Happiness | - | -0.63 (-0.97, -0.30) | < 0.001 |
| GAD-2 >= 3 | 14.64 (5.14, 41.64) | - | < 0.001 |
| PHQ-2 >= 3 | 5.28 (1.79, 15.54) | - | 0.003 |
| Mental illness score | - | 1.82 (1.19, 2.46) | < 0.001 |
|  |  |  |  |
| *Social well-being* |  | - |  |
| Having a person to talk  to when lonely | 0.58 (0.25, 1.35) | - | 0.209 |
| Having a person to ask for help when in trouble | 0.37 (0.17, 0.80) | - | 0.011 |
| Recreational community participation | 0.24 (0.07, 0.75) | - | 0.014 |
| Ethnic community participation | 0.27 (0.11, 0.66) | - | 0.004 |
| Religious community participation | 0.81 (0.29, 2.23) | - | 0.679 |
| Note.  All regressions were adjusted for age, gender, location of residence, months residing in Korea, monthly income, employment status, employment industry type, educational attainment, house size, housing quality, number of cohabitants, marital status, country of origin, and religion. Uncorrected p values were presented.  OR, odds ratio; CI, confidence intervals, GAD, Generalized Anxiety Disorder; PHQ, Patient Health Questionnaire. | | | |

**Supplementary Table 3** Associations of the undocumented status with well-being outcomes after adjusting for potential confounders including outcomes in other well-being categories

|  | Undocumented vs. Documented | | |
| --- | --- | --- | --- |
|  | OR (95% CI) | $\beta$ (95% CI) | p-value |
| *Physical well-being* |  |  |  |
| Self-rated health (average or worse) | 1.36 (0.56, 3.29) | - | 0.491 |
| Occupational injury | 1.65 (0.70, 3.94) | - | 0.255 |
|  |  |  |  |
| *Health behavior* |  |  |  |
| Cigarette smoking | 2.10 (1.06, 4.14) | - | 0.032 |
| Alcohol consumption level |  |  |  |
| – moderate vs. none | 1.76 (1.04, 2.99) | - | 0.035 |
| – heavy vs. none | 4.50 (1.30, 15.54) | - | 0.018 |
| Irregular meal pattern | 1.01 (0.56, 1.82) | - | 0.968 |
|  |  |  |  |
| *Psychological well-being* |  |  |  |
| Happiness | - | -0.55 (-0.79, -0.31) | < 0.001 |
| GAD-2 >= 3 | 5.47 (2.88, 10.39) | - | < 0.001 |
| PHQ-2 >= 3 | 3.64 (1.86, 7.11) | - | < 0.001 |
| Mental illness score | - | 1.47 (1.02, 1.92) | < 0.001 |
|  |  |  |  |
| *Social well-being* |  | - |  |
| Having a person to talk to when lonely | 1.65 (0.84, 3.25) | - | 0.147 |
| Having a person to ask for help when in trouble | 1.39 (0.76, 2.52) | - | 0.282 |
| Recreational community participation | 0.28 (0.13, 0.59) | - | < 0.001 |
| Ethnic community participation | 0.43 (0.23, 0.80) | - | 0.008 |
| Religious community participation | 0.61 (0.31, 1.17) | - | 0.137 |
| Note.  All regressions were adjusted for age, gender, location of residence, months residing in Korea, monthly income, employment status, educational attainment, house size, housing quality, number of cohabitants, marital status, country of origin, and religion. Uncorrected p values were presented.  OR, odds ratio; CI, confidence intervals, GAD, Generalized Anxiety Disorder; PHQ, Patient Health Questionnaire. | | | |

**Supplementary Table 4** Associations of the undocumented status with well-being outcomes with alternative forms of categorization after adjusting for potential confounders

|  | Undocumented vs. Documented | |
| --- | --- | --- |
|  | OR (95% CI) | p-value |
| Self-rated health |  |  |
| – fairly good vs. very good | 1.56 (0.93, 2.62) | 0.092 |
| – average vs. very good | 2.05 (0.81, 5.18) | 0.131 |
| Cigarette smoking |  |  |
| – former smoker vs. never-smoker | 0.79 (0.22, 2.82) | 0.716 |
| – 10 or less/day vs. never-smoker | 1.92 (0.88, 4.17) | 0.101 |
| – 11–20/day vs. never-smoker | 2.21 (0.94, 5.19) | 0.069 |
| – 21–30/day vs. never-smoker | 3.58 (0.81, 15.90) | 0.093 |
| Having a person to talk to when lonely |  |  |
| – disagree vs. strongly disagree | 0.19 (0.04, 0.81) | 0.024 |
| – agree vs. strongly disagree | 0.18 (0.04, 0.71) | 0.014 |
| – strongly agree vs. strongly disagree | 0.29 (0.05, 1.57) | 0.150 |
| Having a person to ask for help when in trouble |  |  |
| – disagree vs. strongly disagree | 0.27 (0.07, 1.02) | 0.054 |
| – agree vs. strongly disagree | 0.24 (0.07, 0.85) | 0.027 |
| – strongly agree vs. strongly disagree | 0.16 (0.04, 0.72) | 0.017 |
| Recreational community participation |  |  |
| – formerly participated vs. never participated | 0.19 (0.08, 0.43) | < 0.001 |
| – currently participating vs. never participated | 0.22 (0.07, 0.67) | < 0.001 |
| Ethnic community participation |  |  |
| – formerly participated vs. never participated | 0.37 (0.20, 0.70) | < 0.001 |
| – currently participating vs. never participated | 0.39 (0.20, 0.77) | < 0.001 |
| Religious community participation |  |  |
| – formerly participated vs. never participated | 0.50 (0.24, 1.03) | 0.061 |
| – currently participating vs. never participated | 0.55 (0.27, 1.14) | 0.109 |
| Note.  All regressions were adjusted for age, gender, location of residence, months residing in Korea, monthly income, employment status, educational attainment, house size, housing quality, number of cohabitants, marital status, country of origin, and religion. Uncorrected p values were presented.  OR, odds ratio; CI, confidence intervals. | | |
